# Supplementary material for: Multifaceted Effect of Rubus Occidentalis on Hyperglycemia and Hypercholesterolemia in Mice with Diet-Induced Metabolic Diseases
Source: Nutrients. 2018 Dec 1;10(12):1846. doi: 10.3390/nu10121846 (PMC6315456; doi:10.3390/nu10121846)
Supplement: Supplementary file 1 [file nutrients-10-01846-s001.pdf]

nutrients

MDPI

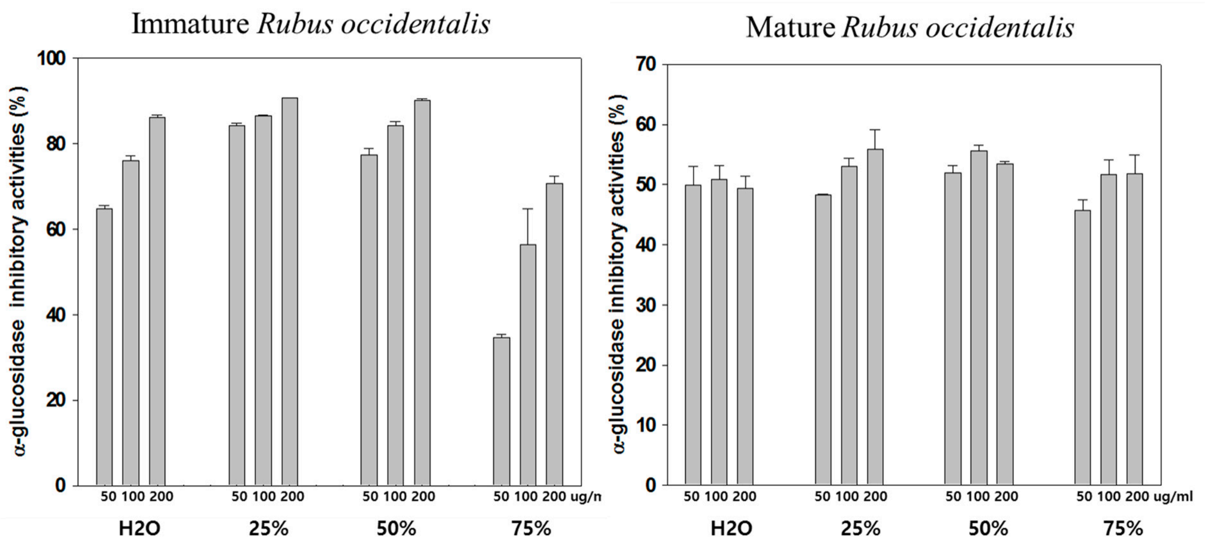

Figure S1. Effects of differently extracted RO forms on alpha-glucosidase inhibitory activities. Various extraction forms of immature and mature RO were tested to determine their alpha-glucosidase inhibitory activities using several concentrations (50, 100, and 200 μg/mL). The alpha-glucosidase inhibitory activity of immature RO was superior to that of ripe fruit, especially at 200 μg/mL, in extraction conditions of water and in 25% and 50% ethanol.

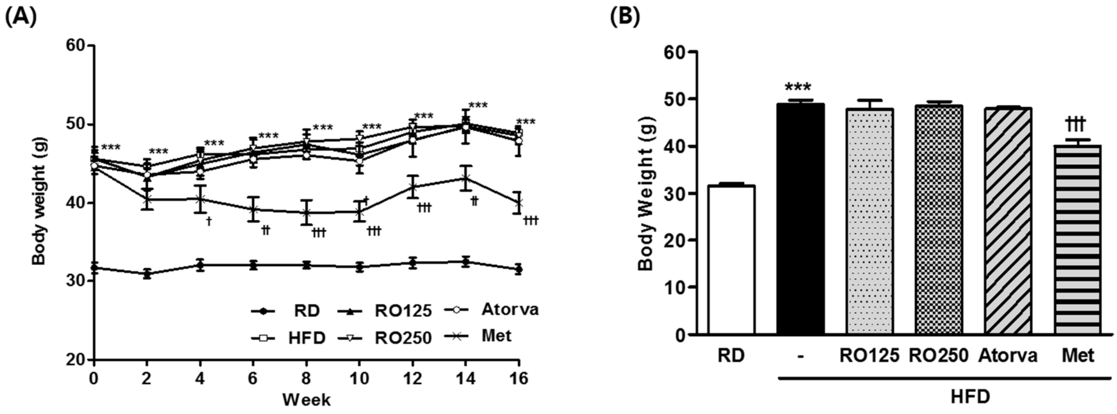

Figure S2. Effect of aqueous extracted RO on body weight change. DIMD mice were fed a HFD diet with RO or control drugs (Atorvastatin and Metformin) for 16 weeks. The changes in body weights (A) were observed every two weeks and the value from the final week are shown in Figure S2B. RD: regular diet-fed mice, HFD: high fat diet-fed mice, HFD + RO 125: HFD-fed mice treated with a low dose (mg/kg/day) of aqueous extract of *Rubus occidentalis* (RO) 125, HFD + RO 250: HFD-fed mice treated with a high dose (mg/kg/day) of RO 250. HFD + Atorva: HFD-fed mice treated with atorvastatin (10 mg/kg/day). HFD + Met: HFD-fed mice treated with metformin (250 mg/kg/day). Values are presented as means ± S.E.M. (n = 7, each group), *** $p < 0.0001$ compared with RD mice. $^{\dagger} p < 0.05$, $^{\dagger\dagger} p < 0.01$, $^{\dagger\dagger\dagger} p < 0.0001$ compared with HFD mice.

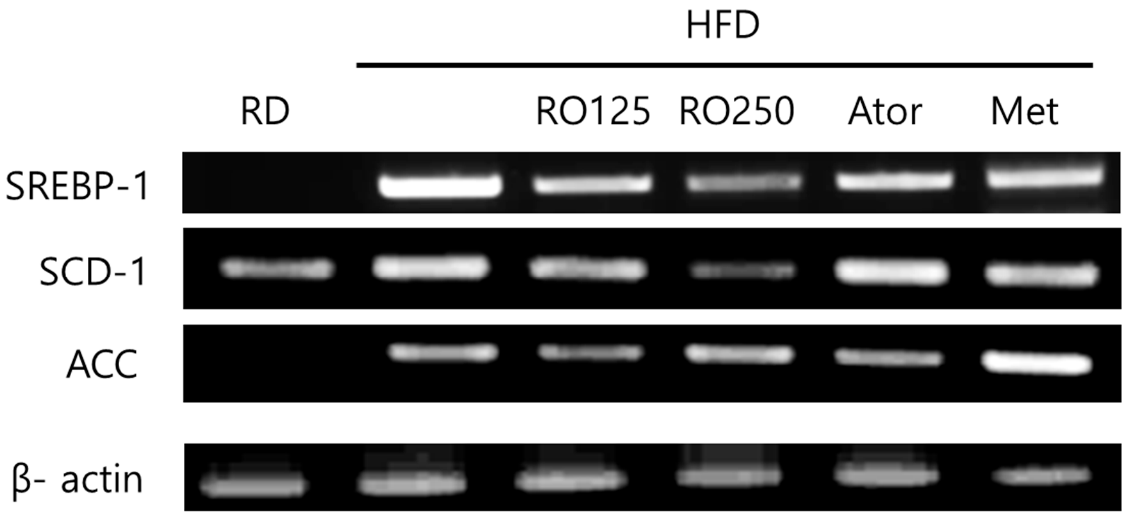

Figure S3. Effects of aqueous extracted RO on RNA expression of genes related to hepatic lipogenesis. The mRNA expression levels of genes related to lipid synthesis and cholesterol metabolism in liver tissue samples were measured by reverse transcription polymerase chain reaction (PCR). RD: regular diet-fed mice, HFD: high fat diet-fed mice, HFD + RO 125: HFD-fed mice treated with a low dose (mg/kg/day) of aqueous extract of *Rubus occidentalis* (RO) 125, HFD + RO 250: HFD-fed mice treated with a high dose (mg/kg/day) of RO 250. HFD + Atorva: HFD-fed mice treated with atorvastatin (10 mg/kg/day). HFD + Met: HFD-fed mice treated with metformin (250 mg/kg/day). The analysis for each gene was performed in triplicate and similar results were obtained in all three experiments. (SREBP-1: sterol regulatory element binding protein 1, SCD-1: stearoyl-CoA desaturase 1, ACC: acetyl CoA carboxylase).
